# Evaluating the Restoration of External Root Resorption Under Biomechanical Stress: A Finite Element Analysis

**DOI:** 10.7759/cureus.71238

**Published:** 2024-10-11

**Authors:** Sevval Celebi, Hesna Sazak Ovecoglu

**Affiliations:** 1 Department of Endodontics, Marmara University, Istanbul, TUR

**Keywords:** biodentine, external root resorption, finite element analysis, gutta-percha, mineral trioxide aggregate

## Abstract

Background: Root resorption is a complex problem in endodontic treatment that considerably affects the structural integrity of teeth. This study aims to assess the biomechanical efficacy of various restorative materials and approaches in treating external root resorption, emphasizing their capacity to improve stress resistance and guarantee long-term success.

Methods: This research employed finite element analysis (FEA) to assess stress distribution in mandibular premolars with external root resorption. Eight models, demonstrating apical and mid-level resorption, were repaired using mineral trioxide aggregate (MTA), Biodentine, or a mixture of these materials with gutta-percha. In order to evaluate each technique's biomechanical performance, the models were exposed to both vertical and oblique stresses.

Results: The findings demonstrated that complete canal obturation with Biodentine or MTA greatly lowered stress levels, with Biodentine exhibiting a marginal advantage. The hybrid approach utilizing gutta-percha produced elevated stress concentrations, especially under oblique loading conditions.

Conclusion: MTA and Biodentine are effective in enhancing resistance to biomechanical forces in teeth with external resorption. However, the combination of gutta-percha with these materials reduces resistance, especially under oblique forces. These findings highlight the importance of material selection in restoring resorbed teeth and improving clinical outcomes.

## Introduction

Endodontic treatment is focused on preserving the tooth's natural functionality by treating a variety of dental pathologies, such as root resorption, which has the potential to limit the treatment's effectiveness [1]. Root resorption is defined by the abnormal degradation and loss of tooth structure, both internally and externally [1]. Not only do these processes impair the tooth's structural integrity, but they also pose substantial obstacles to endodontic procedures, including loss of tooth structure, compromised access, communication pathways to periradicular tissues, unpredictable canal anatomy, and the need for advanced techniques, which make root resorption particularly challenging to accurately diagnose and manage [1,2]. Additionally, the formation of a communication pathway between the root canal system and periradicular tissues is a significant complication in cases of extensive root resorption; this pathway may lead to the extrusion of irrigation and filling materials into the periodontal space [3,4].

Furthermore, the greater likelihood of tooth fracture is an additional critical concern associated with root resorption, which may ultimately lead to tooth loss [2]. The amount of tooth structure that is still present has a significant impact on both long-term retention and fracture resistance. In instances of severe external root resorption, the structural integrity of the tooth is considerably undermined, thereby requiring the utilization of materials that possess the capacity to endure occlusal forces [5]. Gutta-percha has been utilized historically as a filling material. Nevertheless, there have been significant developments in the field in recent years, such as the utilization of thermoplastic gutta-percha and hybrid techniques for root canal obturation. All of these advancements have improved the efficacy of sealing and the overall success rate of endodontic procedures [6]. The 2022 study conducted by Patel et al. examined a case of external root resorption in a maxillary incisor as a case study. The existence of external inflammatory resorption in teeth 21 and 22 was confirmed by cone-beam computed tomography (CBCT) scans and periapical radiography. Teeth 21 and 22 underwent root canal treatment, with the former being obturated with gutta-percha and the latter with Biodentine. Significant bone healing was observed in the surrounding area of both teeth in follow-up images [1].

Historically, many procedures and materials have been employed to address perforated root resorption. Calcium hydroxide and surgical procedures are among the most commonly employed alternatives and have demonstrated efficacy. Calcium hydroxide-based recalcification techniques have traditionally been favored; however, their use comes with certain disadvantages. One of the most notable limitations is the need for multiple appointments, requiring repeated applications over a long period to achieve hard tissue closure of root perforations [7].

In contrast, mineral trioxide aggregate (MTA) has become increasingly recognized in surgical repairs for its biocompatibility, exceptional sealing capabilities, and capacity to enhance osteogenesis and cementogenesis [8]. In addition to MTA, calcium silicate cement formulations, such as Biodentine, are becoming known as promising options for dentin restoration and endodontic applications. Biodentine offers several advantages over MTA, such as greater mechanical stability, a reduced setting time, and better handling [9]. With these qualities, Biodentine becomes a vital tool for endodontic treatments, allowing for the optimization of procedures and the improvement of patient results.

The computational technique known as finite element analysis (FEA) is used to study complicated structures and forecast the possibility of their failure under certain circumstances [10]. There are a lot of benefits to using FEA instead of laboratory testing. One of them is that it allows you to manipulate factors and analyze stress in the root canal wall, which is impossible to do with traditional methods. In comparison to conventional fragility tests, FEA provides a more comprehensive stress analysis, making it the preferable method for assessing stress in dental biomechanics. This is essential for the optimization of treatment protocols, the improvement of our understanding of mechanical behavior in biomedical structures, and the evaluation of tooth and tissue layers [11].

This study aims to evaluate the effect of restorations on stress distribution under horizontal and oblique forces on a mandibular second premolar with simulated external root resorption, utilizing three-dimensional FEA. The study's null hypothesis proposes that Biodentine, due to its superior biomechanical features, will improve restorative effectiveness by enhancing stress distribution under biomechanical stresses in comparison with MTA.

This article was previously presented as a meeting abstract at the 2024 IFEA World Endodontic Congress on September 14, 2024.

## Materials and methods

Developing models

The ANSYS Spaceclaim software (ANSYS, Inc., Canonsburg, USA) was employed to generate models of mandibular premolar teeth, their supporting tissues, and external resorption cavities within the root canals. The measurements of the surrounding tissues and the morphology of the tooth models were obtained from the existing literature [12,13]. Eight different restored tooth models were developed including enamel, dentine, composite filling, cortical bone, spongy bone, periodontal ligament (PDL), gutta-percha filling, MTA, and Biodentine. The dimensions of the external resorption cavities were 1.8 mm (cervical-apical) x 1.43 mm (buccal-lingual) in the apical area and 2.8 mm (cervical-apical) x 1.72 mm (buccal-lingual) in the middle root area. The distances from the center of the resorption cavities to the root apices were 3 mm in the apical area and 8 mm in the middle region.

Model E1: The resorption cavity was located in the apical region of the root. MTA was used to fill the resorption cavity and the lumen beneath the canal. The lumen above the canal was filled with gutta-percha using a hybrid technique.

Model E2: The resorption cavity was located in the apical region of the root. The complete root canal was filled with MTA.

Model E3: The resorption cavity was located in the apical region of the root. Biodentine was used to fill the resorption cavity and the lumen beneath the canal. The lumen above the canal was filled by gutta-percha using a hybrid technique.

Model E4: The resorption cavity was located in the apical region of the root. The root canal was completely filled with Biodentine.

Model E5: The resorption cavity was located in the middle section of the root. MTA was used to fill the resorption cavity and the lumen beneath the canal. The lumen above the canal was filled by gutta-percha using a hybrid technique.

Model E6: The resorption cavity was located in the middle section of the root. The complete root canal was filled with MTA.

Model E7: The resorption cavity was located in the middle section of the root. Biodentine was used to fill the resorption cavity and the lumen beneath the canal. The lumen above the canal was filled by gutta-percha using a hybrid technique.

Model E8: The resorption cavity was located in the middle section of the root. The root canal was completely filled with Biodentine.

The models were constructed with resorption cavities at the apical and middle levels, and distinct filling techniques were employed using the monoblock obturation technique with MTA or Biodentine, along with a hybrid technique that combined gutta-percha with MTA or Biodentine. The resorptions were defined as unilateral and exclusively external. The access cavity was restored virtually using software by composite resin. Mathematical models were developed by dividing geometric models into simple, smaller components known as meshes.

FEA

The geometric models were prepared, and a finite element mesh was manually superimposed onto them. The points where the sides of components meet are referred to as "nodes," and these are the points where elements are joined. Table 1 provides the following quantities of elements and nodes for various analysis models. The first step in the finite elements solution technique is the discretization of the solution domain and the selection of element type. The ANSYS Workbench software (ANSYS, Inc.) was used to mathematically construct and prepare the models for analysis after the modeling procedure was completed in the ANSYS SpaceClaim software. The second phase involves the assignment of material properties to respected elements. All materials were assumed to be linearly elastic, isotropic, and homogeneous. Poisson's ratio and Young's modulus were derived from the literature and are summarized in Table 2 [14,15]. The final phase involves the definition of loading conditions and the introduction of boundary conditions. To conduct the analyses, the mathematical models that were generated in ANSYS Workbench were converted to the LS-DYNA solver (ANSYS, Inc.). All eight models were subjected to stress distribution testing.

**Table 1:** The table presents the quantities of elements and nodes associated with different analysis models.

Model	Total number of nodes	Total number of elements
Model 1/3	254,162	1,009,130
Model 2/4	257,019	1,021,833
Model 5/7	256,665	1,016,680
Model 6/8	255,653	1,013,645

**Table 2:** The values for Poisson's ratio and Young's modulus have been obtained from the literature are presented. MTA: mineral trioxide aggregate

Material	Elastic modulus (MPa)	Poisson's ratio
Cortical bone	13700	0.30
Spongy bone	1370	0.30
Dentin	18600	0.31
Enamel	41000	0.31
Periodontal ligament	68.9	0.45
Gutta-percha	140	0.45
Resin composite	12000	0.30
MTA	15700	0.23
Biodentine	22000	0.30

Forces of 300 N were applied individually to the tooth's buccal cusp in each model, at angles of 135 and 90 degrees to the tooth's long axis [15]. The forces were distributed among the adjacent nodes to prevent stress singularities in the loading regions. The models were restricted by fixing all degrees of freedom at the nodes positioned in the anterior, posterior, and inferior sections of the bone, so inhibiting movement along all three axes.

Stress distribution analysis

The qualitative results were presented as stress maps, with warm colors indicating the highest stress levels and cool colors denoting the lowest stress values; conversely, the quantitative data were illustrated as distinct stress values. The distribution of Von Mises stress in the enamel, dentin, restoration, and PDL was examined.

## Results

Von Mises stresses were applied during the stress analysis. The numerical values were measured in MPa, and stress concentrations were depicted using a color scale.

Stress generated in the whole tooth

**Figure 1:**
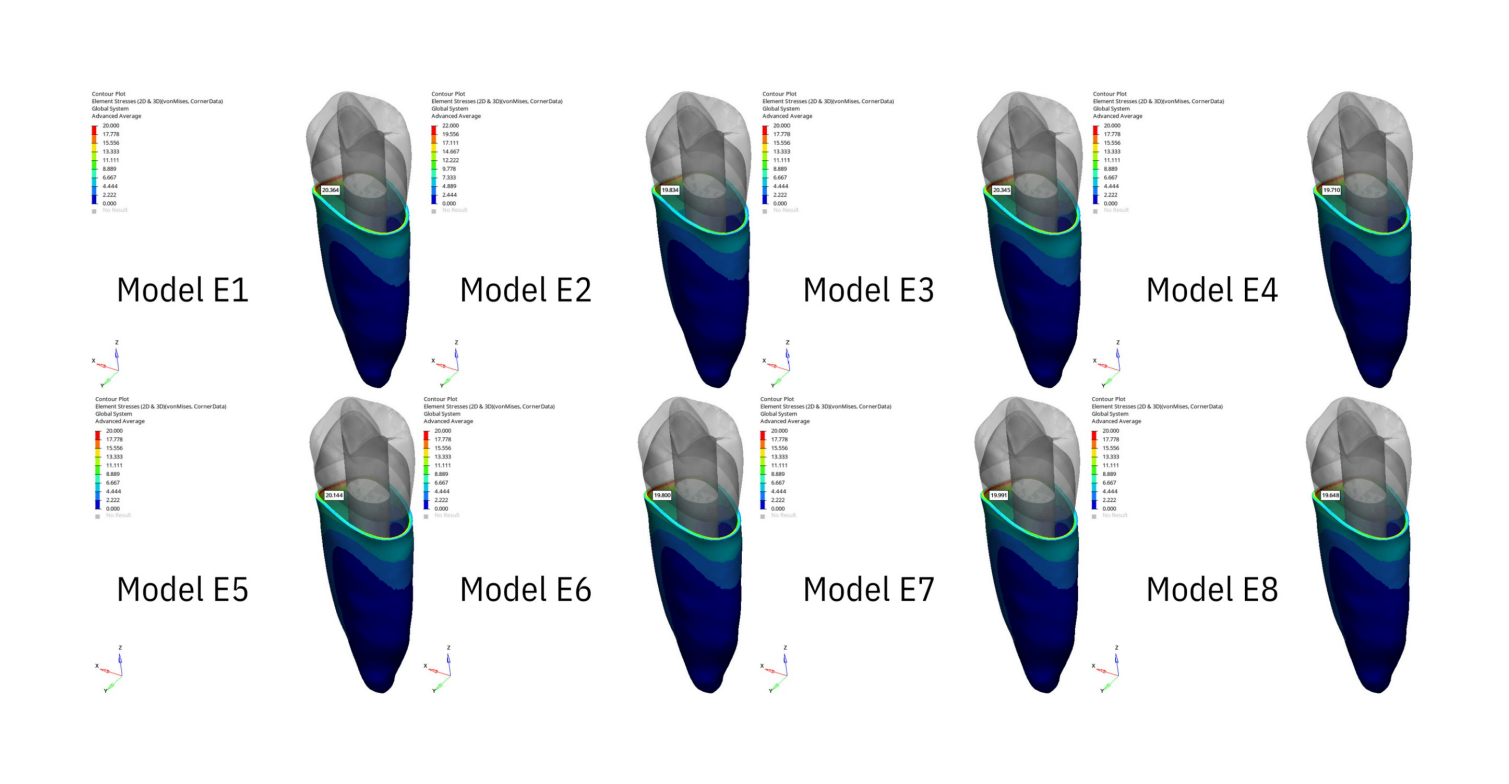
The figure illustrates the areas of stress distribution throughout periodontal ligament resulting from the application of oblique forces.

**Figure 2:**
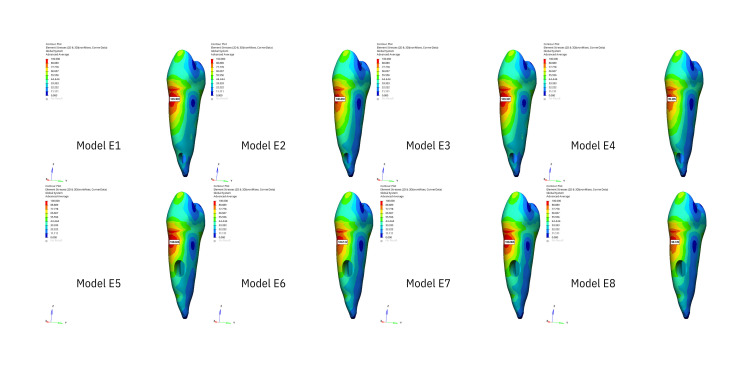
The figure illustrates the areas of stress distribution throughout the entire tooth resulting from the application of oblique forces.

The stress distribution areas in the entire tooth that are the result of the application of oblique forces are illustrated in Figure 1, while the stress distribution areas resulting from vertical forces are illustrated in Figure 2. The greatest stress under oblique forces was observed at the cervicobuccal level in both the apical and middle resorption groups. The buccal face of the buccal tubercle exhibited the second-greatest stress level. The buccal tubercle exhibited the greatest stress level when vertical forces were applied, while the cervicobuccal level exhibited the second-highest stress level. Stress decreased as it approached the apex in both scenarios.

Table 3 contains the maximal stress values. The apical group E3 and the middle group E7 exhibited the highest stress among the oblique forces, while the apical group E4 and the middle group E8 exhibited the lowest stress. The apical group E3 and the middle group E7 generated the maximum stress under vertical forces, while the apical group E4 and the middle group E8 generated the minimum stress.

**Table 3:** The table displays the maximum stress values observed in the periodontal ligament and the entire tooth. ''V'' refers to vertical load; ''O'' refers to oblique load; PDL: periodontal ligament

Models	Model E1		Model E2		Model E3		Model E4		Model E5		Model E6		Model E7		Model E8	
Maximum von Mises stresses (MPa)	V	O	V	O	V	O	V	O	V	O	V	O	V	O	V	O
Whole Tooth	95.524	109.309	95.515	100.892	95.534	109.331	95.506	99.385	95.092	108.026	95.088	100.122	95.101	108.066	95.079	98.720
PDL	6.076	20.364	5.992	19.834	6.071	20.345	5.948	19.710	6.038	20.144	5.976	19.800	6.017	19.991	5.942	19.648

Stress generated in PDL

**Figure 3:**
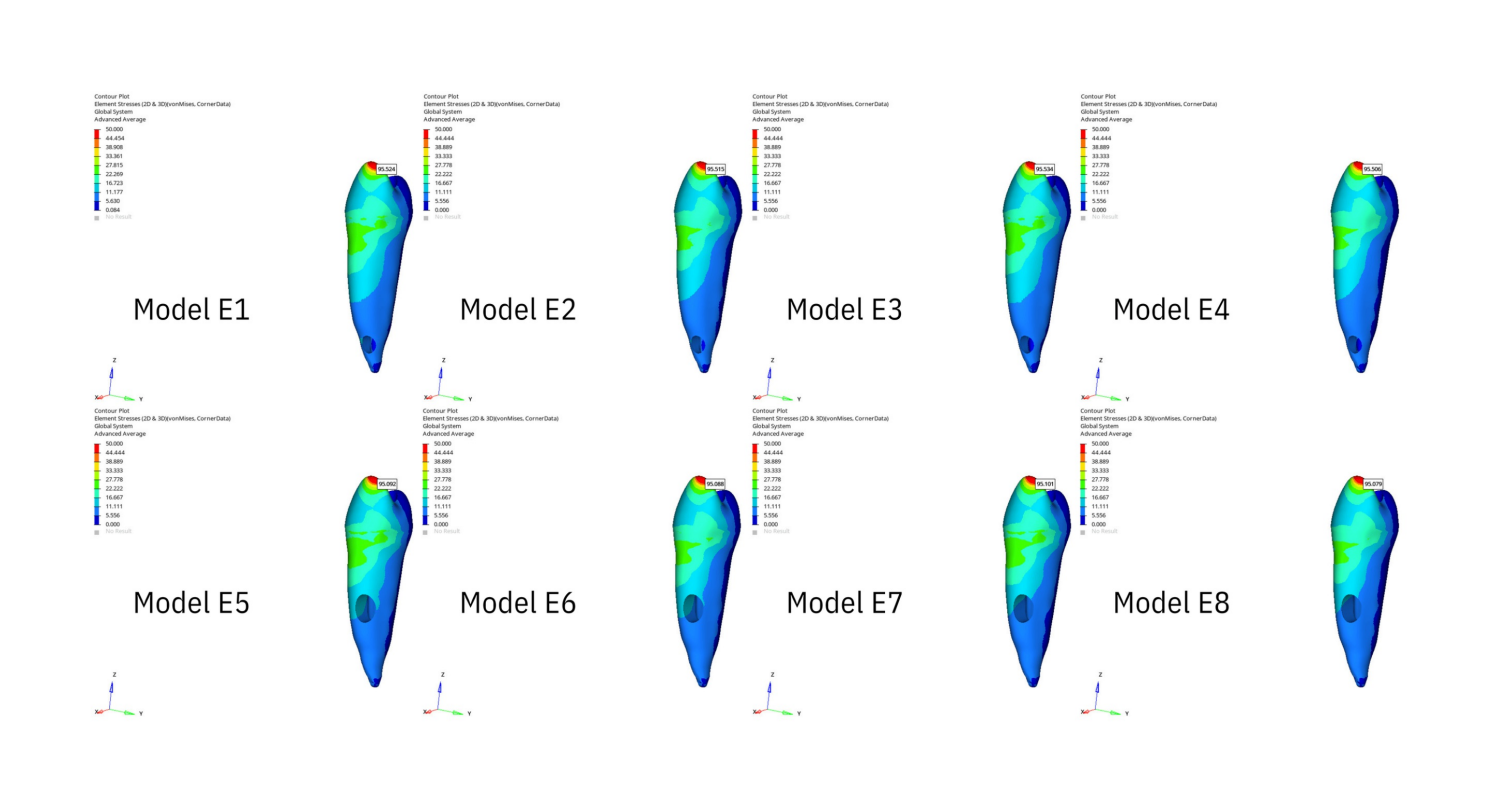
The figure illustrates the areas of stress distribution throughout the entire tooth resulting from the application of vertical forces.

**Figure 4:**
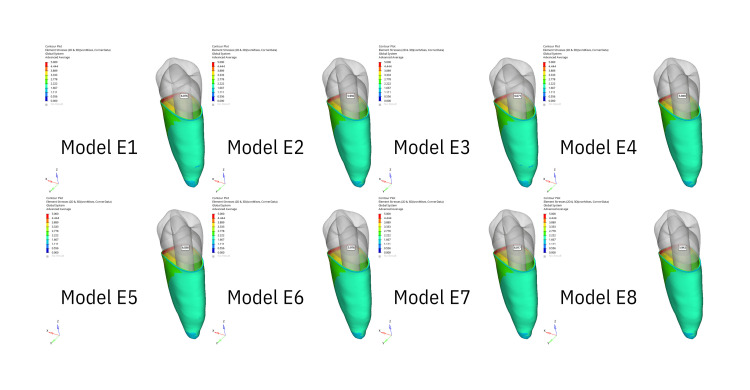
The figure illustrates the areas of stress distribution throughout periodontal ligament resulting from the application of vertical forces.

The stress distribution within the PDL, affected by vertical stresses illustrated in Figure 3 and oblique forces, is shown in Figure 4. The buccal face and cervical level of the tooth were the locations where the maximal stress level was observed at the starting point of the PDL in all groups. Stress decreased apically under both pressures.

Table 3 presents the highest stress values recorded in the PDL. The apical group (E1) and middle group (E5) exhibited the highest stress among oblique forces, whereas the apical group (E4) and middle group (E8) had the lowest stress. Under vertical forces, the maximum stress occurred in the apical group (E1) and the middle group (E5), whereas the smallest stress was seen in the apical group (E4) and the middle group (E8).

## Discussion

Root resorption, a pathological process that is characterized by the loss of dental hard tissue as a result of odontoclastic activity, has the potential to substantially weaken the tooth structure, thereby increasing its susceptibility to fractures [2]. This issue may impact either the root canal or the external surface of the root in permanent teeth [1]. The restoration of tooth integrity and fracture prevention need the careful selection of materials and processes [16]. A treatment strategy must be formulated individually, considering the patient's personal characteristics alongside the clinical and radiological data. When a tooth is considered restorable, endodontic intervention may be necessary, either orthograde or surgical [1]. The restoration of resorption cavities is a considerable difficulty, especially when significant challenges form a connection between the tooth and the periodontium via the cementum [4]. Due to the variety of instances, it is crucial that treatment planning and material selection be executed with precision and customized to the unique characteristics and requirements of each specific case [17].

The potential of materials such as MTA and Biodentine has been investigated in order to determine their suitability for addressing root resorption and filling the residual canal, taking into account all of the prior factors. Exceptional sealing capabilities and biocompatibility are the primary reasons why MTA is frequently chosen for successful restoration in resorption cases. MTA establishes itself as a reliable option in clinical contexts by forming a durable and long-lasting barrier, reducing the likelihood of additional bone destruction, and promoting epithelial proliferation. MTA does have certain drawbacks, though, such as the possibility of tooth discoloration and handling difficulties. In addition, the complete removal of MTA from the root canal space when it is employed as a root filling is a difficult task [8].

In contrast, Biodentine provides better color stability and ease of use and is also highly biocompatible. It forms an efficient seal with a lower chance of tooth discoloration since it sets more quickly and adheres to dentin well [8]. The inability to remove Biodentine during retreatment due to the lack of a recognized solvent is one of its drawbacks [18]. Although both materials have been shown to be effective, Biodentine may provide certain benefits in terms of its simplicity of use and shorter treatment times. In spite of this, MTA continues to exhibit exceptional dependability, particularly in terms of long-term results. Thus, each material has unique advantages and disadvantages in clinical practice, and the selection should be determined by the patient's unique needs and treatment circumstances.

In order to guarantee the optimal distribution of biomechanical stress, it is of the highest priority to ensure the effective management of obturation of the remaining canal space upon the finalization of the resorption cavity repair. Nonetheless, there is no agreement on the most appropriate material for this function [4]. The canal space can be completely filled with either MTA or Biodentine. Alternatively, the residual area may be filled with a mixture of gutta-percha and sealer following the application of MTA or Biodentine, referred to as a hybrid approach. This method integrates the sealing capabilities of bioactive cement with the adaptability of gutta-percha [5].

While gutta-percha, which is often used in combination with a sealer, shows a decreased tendency to discolor and improved workability, it may not always provide the same level of sealing effectiveness as MTA or Biodentine, especially when dealing with intricate canal anatomy [19]. The selection of material should be guided by the specific clinical scenario and intended outcomes, as each material has unique strengths and limitations. The hybrid technique, which integrates gutta-percha with MTA or Biodentine, offers a balanced approach to biomechanical stress management and sealing [8]. On the other hand, additional research is necessary to establish definitive guidelines for the optimal selection of materials.

This study aimed to evaluate the resilience of various root canal filling materials and techniques to provide guidance for clinical applications. The analysis of dental materials and tissues was conducted using FEA, a method that accurately simulates real-world conditions through numerical analyses that would be difficult to perform in vivo. The procedure involves creating a mesh of the geometry of the structure, which facilitates the simplification of complex equation resolution. The mesh elements serve as the discretization of the physical model, with each element characterized by the mechanical properties of the constituent materials. The distribution of stresses is illustrated through color maps on a predetermined scale, which is founded on established failure criteria [20]. A standardized model of a single-rooted lower second premolar was developed in this study, respecting the dimensions documented in the literature. The purpose of this action was to achieve standardization and facilitate a balanced evaluation of the restoration through the application of FEA. The restoration's durability was assessed in two specific areas: the apical region, known for being prone to resorption, and the mid-root level [21].

The lower premolar root was designed with a total length of 14.5 mm, divided into sections of approximately 4.8 mm each, representing one-third of the overall root length. The resorption cavities were designed with diameters measuring 1.8 mm at the apical level and 2.8 mm at the mid-root level, ensuring uniform support from the surrounding dentin. During the construction of these models, the canal space was completely filled with either MTA or Biodentine. Alternatively, after the resorption cavity was restored with MTA or Biodentine, the remaining space was filled with a combination of gutta-percha and resin-based sealer.

Subsequently, the models were exposed to 300 N oblique or vertical occlusal stresses to assess the endurance of the employed repair techniques and materials. The results indicated that the application of Biodentine along the whole canal in cases of apical or mid-root resorption produced the least stress under both vertical and oblique forces, demonstrating a significant difference compared to MTA. The null hypothesis is partially accepted based on these results. Although both Biodentine and MTA demonstrated satisfactory performance, the results indicate that Biodentine does affect restorative efficacy to a significant degree. This discovery supports prior research that established Biodentine's better stress distribution capabilities. To considerably improve fracture resistance and successfully reinforce weak dental structures, Arıcan et al. (2022) revealed that the most effective material for restoring external cervical resorption cavities is Biodentine [22]. Nevertheless, MTA outperformed Biodentine in the fracture resistance testing carried out for the Bayram and Bayram study [23].

The initial properties of MTA and Biodentine are comparable; however, their performance differs due to variations in elastic moduli post-setting. Biodentine, upon setting, exhibits an elastic modulus that closely resembles that of dentin. The elastic modulus of dentin ranges from approximately 14 to 18.6 GPa, whereas the elastic modulus of Biodentine, comparable to MTA, is about 15 to 30 GPa after two weeks. The enhanced stress distribution and adhesion properties of Biodentine may be attributed to its closer similarity to dentin; however, the superior fracture strength noted with MTA in the fracture resistance tests may have resulted from other factors, such as its long-term interaction with the tooth structure or the specific testing conditions [23]. In similar circumstances, the complete filling of the canal and resorption cavity with MTA exhibited low-stress formation, although no significant difference was observed when compared to Biodentine. This conclusion is supported by Kabtoleh et al. (2023), who found that bioceramic putty and MTA successfully improve overall tooth strength by restoring fracture resistance in endodontically treated molars with simulated strip perforation [24]. In contrast, the hybrid technique, which involves the filling of the resorption cavity with biocompatible materials and the subsequent filling of the remaining irregularly shaped canal with gutta-percha, exhibited the highest stress accumulation under forces applied in both directions. This outcome is in accordance with the findings of Türker et al. (2018), who noted that the fracture resistance was higher when backfilling with calcium silicate-based cements (CSCs) was used in comparison to a gutta-percha/sealer combination [4]. It is important to observe that the combination of gutta-percha with Biodentine resulted in a higher stress than the combination with MTA. This disparity may be attributed to the insignificant difference in the elastic modulus between gutta-percha and dentin. This discovery is also in accordance with the findings of Li et al. (2006), who emphasized the significance of utilizing dental materials with an elastic modulus that is closely aligned with that of dentin to reinforce the tooth structure [25].

In both scenarios, the root's cervicobuccal level encountered the greatest amount of stress. This location received the most stress from both oblique and vertical forces, making it a significant spot for potential structural problems. The high-stress concentration in this location suggests that the root's cervicobuccal area is particularly vulnerable to damage or degeneration over time, especially under continuous loading. This finding is aligned with the findings of Baghani et al. (2023), who also discovered that the buccal region of lower molars is more prone to stress concentration [26].

The group in which the whole canal and resorption cavity were filled with Biodentine exhibited the lowest stress levels in the PDL under oblique and vertical stresses. Consequently, the group composed exclusively of MTA demonstrated no notable difference from the preceding group. The peak stress levels in the PDL, arising from both oblique and vertical forces, were noted in the hybrid approach where the resorption cavity was filled with MTA and the remaining canal with gutta-percha, regardless of the resorption's position. A decrease in peak stress values was detected towards the apical portion of the PDL, as evidenced by the distribution patterns of stress seen across it, which were influenced by both vertical and oblique forces. This finding is consistent with the studies of Bucchi et al. (2022), who also observed tension distribution patterns in the PDL of teeth reinforced with dentin and cementum [27]. Furthermore, this discovery is in accordance with the understanding that stress levels are generally lower in the PDL than in the root, with the maximum stress concentrations occurring at the buccal surface and cervical level.

The analysis of stresses induced by oblique and vertical forces demonstrated that oblique forces produced more stress accumulation. This finding aligns with the data of Maceri et al. (2010), who indicated that oblique loads present a higher risk than vertical loads in endodontically treated teeth [28].

Even though FEA is a useful tool for understanding how teeth are loaded biomechanically, it fails to fully duplicate the complexity of clinical situations that happen in real life. In clinical practice, tooth fractures often arise from dynamic loads that are challenging to recreate experimentally. Moreover, the materials utilized in this work were assumed to be isotropic, homogeneous, and linearly elastic, while living tissues are inherently anisotropic and heterogeneous. Thus, the virtual simulation failed to capture the full complexity of the real clinical environment, including the presence of flexural strength. Moreover, FEA neglects essential oral environmental elements, including microleakage and microbial contamination, hence, limiting its focus to a strictly biomechanical approach [29]. Thus, establishing direct clinical connections is difficult, as treatment outcomes are affected by numerous factors outside biomechanical considerations.

## Conclusions

Within the limitation of this study, the results indicate that MTA and Biodentine exhibit comparable performance in the repair of external resorption, thereby increasing resistance to biomechanical forces. Nevertheless, the restoration's resistance to biomechanical stress declines when gutta-percha is combined with either material, particularly when the restoration is subjected to oblique loading. In addition, future research should examine the long-term clinical results of a variety of restorative materials in simulated oral conditions, as the success of these restoration techniques in clinical settings may be influenced by the interplay of biomechanics, material properties, and biological factors.
